# Predictors of Acute Kidney Injury (AKI) among COVID-19 Patients at the US Department of Veterans Affairs: The Important Role of COVID-19 Vaccinations

**DOI:** 10.3390/vaccines12020146

**Published:** 2024-01-30

**Authors:** Lilia R. Lukowsky, Claudia Der-Martirosian, Heather Northcraft, Kamyar Kalantar-Zadeh, David S. Goldfarb, Aram Dobalian

**Affiliations:** 1 Veterans Emergency Management Evaluation Center (VEMEC), US Department of Veterans Affairs, North Hills, CA 91343, USA; claudia.der-martirosian@va.gov (C.D.-M.); heather.northcraft@va.gov (H.N.); aram.dobalian@va.gov (A.D.); 2 The Lundquist Institute for Biomedical Innovation, Harbor UCLA Medical Center, Torrance, CA 90502, USA; kamyar.kalantar-zadeh@va.gov; 3 Tibor Rubin VA Medical Center, Long Beach VA Healthcare System, Long Beach, CA 90822, USA; 4 New York Harbor VA Healthcare System (NYHHS), US Department of Veterans Affairs, New York, NY 10010, USA; david.goldfarb@va.gov; 5 NYU Langone Health, NYU Grossman School of Medicine, New York, NY 10016, USA; 6 Division of Health Services Management and Policy in the College of Public Health, The Ohio State University, Columbus, OH 43210, USA

**Keywords:** COVID-19, pandemic, Veterans, acute kidney injury, COVID-19 vaccinations

## Abstract

Background: There are knowledge gaps about factors associated with acute kidney injury (AKI) among COVID-19 patients. To examine AKI predictors among COVID-19 patients, a retrospective longitudinal cohort study was conducted between January 2020 and December 2022. Logistic regression models were used to examine predictors of AKI, and survival analysis was performed to examine mortality in COVID-19 patients. Results: A total of 742,799 veterans diagnosed with COVID-19 were included and 95,573 were hospitalized within 60 days following COVID-19 diagnosis. A total of 45,754 developed AKI and 28,573 AKI patients were hospitalized. Use of vasopressors (OR = 14.73; 95% CL 13.96–15.53), history of AKI (OR = 2.22; CL 2.15–2.29), male gender (OR = 1.90; CL 1.75–2.05), Black race (OR = 1.62; CL 1.57–1.65), and age 65+ (OR = 1.57; CL 1.50–1.63) were associated with AKI. Patients who were vaccinated twice and boosted were least likely to develop AKI (OR = 0.51; CL 0.49–0.53) compared to unvaccinated COVID-19 patients. Patients receiving two doses (OR = 0.77; CL = 0.72–0.81), or a single dose (OR = 0.88; CL = 0.81–0.95) were also less likely to develop AKI compared to the unvaccinated. AKI patients exhibited four times higher mortality compared to those without AKI (HR = 4.35; CL 4.23–4.50). Vaccinated and boosted patients had the lowest mortality risk compared to the unvaccinated (HR = 0.30; CL 0.28–0.31). Conclusion: Use of vasopressors, being unvaccinated, older age, male gender, and Black race were associated with post COVID-19 AKI. Whether COVID-19 vaccination, including boosters, decreases the risk of developing AKI warrants additional studies.

## 1. Introduction

The severe acute respiratory coronavirus 2 (SARS-CoV-2), which causes coronavirus disease (COVID-19), has a well-documented association with the development of acute kidney injury (AKI) [1,2,3,4,5,6,7]. This connection emerged from the start of the COVID-19 pandemic as one of the most common non-respiratory complications of COVID-19 infection. It was also shown that more COVID-19 patients developed AKI compared to patients with other respiratory infections such as influenza [1]. Additionally, COVID-19 patients who developed AKI had higher mortality, were more likely to require renal replacement therapy (RRT),and were less likely to recover kidney function [1]. COVID-19 patients who developed AKI were also more likely to show a faster rate of decrease in estimated glomerular filtration rate (eGFR) after being discharged from a hospital stay compared to AKI patients without COVID-19 [3,8,9]; about 30% of surviving COVID-19 patients receiving RRT remained on dialysis after being discharged from a hospital [6].

In addition to RRT [3,4,6], COVID-19 AKI patients also had an increased requirement for mechanical ventilation [1,2,3,4]. This, in turn, has placed increased hardships on hospitals and hospital staff’s ability to provide life-saving treatments [10,11]. Furthermore, these patients also had a decreased likelihood of recovering kidney function after being discharged from a hospital stay [2,3,6]. Patients hospitalized for COVID-19 who developed AKI had both longer hospital stays [2,3] and higher mortality rates [2,3,6,7], compared to COVID-19 hospitalized patients who did not develop AKI. Being male, older, or Black [2,3,4,5] has been associated with developing AKI among COVID-19 patients.

During the early months of the pandemic, a hospital in Washington State reported that 19% of hospitalized COVID-19 patients developed AKI [12]. Meta-analyses and literature review studies have indicated that the prevalence of AKI among COVID-19 hospitalized patients was between 9% and 37% [7,13,14,15,16], with mortality as high as 52% [7]. Similarly, several hospitals in New York City [17] reported that 20–40% of intubated ICU patients required RRT [6,10], while hospital mortality among COVID-19 AKI patients was about 45% [18].

To our knowledge, no study has examined predictors of AKI and mortality among COVID-19-positive VA patients over the 3-year period to ascertain whether and how factors such as vaccinations and sociodemographic and clinical characteristics affected development of AKI and mortality. We conducted a retrospective longitudinal study of a nationwide cohort of VA patients who were diagnosed with COVID-19 between January 2020 and December 2022, and evaluated sociodemographic and clinical characteristics that were associated with the development of AKI, as well as predictors of mortality among COVID-19 patients who developed AKI. We also examined the impact of COVID-19 vaccination as a preventive factor on developing AKI among COVID-19 patients and mortality among COVID-19 VA patients who developed AKI.

## 2. Methods

### 2.1. Study Population, Design, and Exclusion Criteria

A cohort of COVID-19-positive VA patients was identified using electronic health records (EHR) from the VA COVID-19 Shared Data Resource (CSDR), VA Corporate Data Warehouse (CDW) [19]. In the CSDR, a positive COVID-19 diagnosis was defined as having a positive PCR or antigen result, or evidence of COVID-19 positivity reported in clinical notes. Only the antigen results were included from both VA and the community (i.e., outside of the VA). Additionally, COVID-19 diagnosis date was defined as the date for the first positive PCR or antigen COVID-19 test.

We considered AKI diagnosis as a COVID-19 complication if the onset of AKI occurred within 60 days of the COVID-19 diagnosis, which was defined in CSDR as “ever/never diagnosed with AKI between the date of onset of COVID-19 and 60 days post COVID-19”. Patients with a history of end-stage kidney disease (ESKD) were excluded from the study.

### 2.2. Outcomes and Follow-Up

The primary outcome of the study was AKI diagnosis, which was defined as developing AKI within 60 days after being diagnosed with COVID-19. The secondary outcome was all-cause mortality within 60 days from COVID-19 diagnosis among all patients and separately among AKI patients. Dates of death were collected from the CDW VA vital status records.

### 2.3. Analysis

Bivariate chi-square analysis was conducted to examine differences in comorbidities, COVID-19 symptoms, complications, emergency care indicators, lab parameters, and demographic characteristics between COVID-19 AKI and non-AKI patients. Additional analyses were conducted to examine differences in length of stay for hospitalizations (LOS), ICU admissions (ICSLOS) and acute care admissions (ALOS), and hospital readmissions among COVID-19 AKI and non-AKI patients who were hospitalized within 60 days from COVID-19 diagnosis.

Multivariate logistic regression analysis was conducted for all COVID-19 diagnosed patients, as well as for hospitalized COVID-19 patients, to examine predictors of AKI within 60 days from COVID-19 diagnosis. These regression models adjusted for age, gender, race, marital status, BMI, history of AKI, Charlson comorbidity index (CCI), smoking status, geographical region, calendar year of COVID-19 diagnosis, history of diabetes, hypertension and heart disease, presence of cold and digestive COVID-19 symptoms, vaccination status before COVID-19 diagnosis, and use of vasopressors during COVID-19 illness.

Finally, 60-day survival analyses were performed using Cox proportional hazard models, adjusting for the same predictors as mentioned above to determine the differences in mortality risk between AKI and non-AKI COVID-19 patients, using AKI diagnoses as a major predictor of mortality. Additionally, the 60-day analyses were performed on a sub-group of AKI patients to determine which predictors were associated with mortality.

All analyses were performed using SAS Enterprise Guide 7.1 software (SAS Institute, Cary, NC, USA). This study was approved by the VA Greater Los Angeles Institutional Review Board.

## 3. Role of the Funding Source

This study was funded by US Department of Veterans Affairs. Our funders had no role in any aspect of the design, analysis, or conduct of the study. The study was conducted according to the guidelines of the Declaration of Helsinki and approved by the VA Greater Los Angeles Healthcare System Institutional Review Board (Project Number: 1616040. Approval Date: 8 March 2020).

## 4. Results

### 4.1. Patient Characteristics

The study cohort included 742,799 COVID-19 VA patients diagnosed between the 2020 and 2022 calendar years, including 95,573 (12.9%) who were hospitalized within 60 days from day of COVID-19 diagnosis. A total of 45,754 (6.2% of all, 47.9% of hospitalized patients) developed AKI as a complication of COVID-19, and 28,573 (62.4%) of AKI patients were hospitalized within 60 days form COVID-19 diagnosis. Additionally, 1998 AKI patients required renal replacement therapy (RRT), and 1394 developed chronic kidney failure (CKF). The highest number of COVID-19 patients who developed AKI (n = 5124) occurred in January 2022, followed by December 2020 (n = 3429), and August 2021 (n = 2456) with the highest number of AKI patients receiving dialysis in January 2022 (n = 159), followed by January 2021 (n = 156) and December 2020 (n = 149) (Figure S1).

Table 1 illustrates descriptive characteristics including comorbidities, type of COVID-19 symptoms, healthcare utilization (e.g., ER visits, hospitalizations, ICU admissions, use of supplemental oxygen and ventilators), complications associated with COVID-19, and demographic characteristics by AKI status among all and separately among hospitalized COVID-19 patients. Table S1 provides additional indicators examined but not included in Table 1. The mortality rate among AKI patients was 34% (37% among hospitalized AKI patients) compared to 6% among non-AKI patients (17% among hospitalized non-AKI patients). Among AKI patients, 54% had a history of diabetes compared to 27% of non-AKI patients (54% hospitalized AKI vs. 49% hospitalized non-AKI), 57% had history of cardiovascular diseases (CVD) compared to 51% of non-AKI patients (60% vs. 74% hospitalized), and 83% had a history of hypertension compared to 51% of non-AKI patients (84% vs. 71% hospitalized). Additionally, 29% of AKI patients (33% hospitalized) had a Charlson comorbidity index over 5 compared to 7% (20% hospitalized) of non-AKI patients. Indicators such as emergency department (ER) visits, percent hospitalization and rehospitalization, and ICU admissions were significantly higher among AKI patients. Regarding demographic characteristics, 74% (75% hospitalized) of AKI patients were older than 65 years, compared to 39% of non-AKI patients (61% hospitalized), and 27% (29% hospitalized) were Black, compared to 19% (22% hospitalized) of non-AKI patients.

Among hospitalized AKI patients, the average hospital length of stay (LOS) for AKI patients was 11.9 days compared to 7.5 days among non-AKI patients, with an ICU LOS of 9.1 and 6.7 days, respectively (Table 2). Additionally, the average number of days on a ventilator was 10.8 for AKI patients compared to 8.1 for non-AKI patients.

Baseline creatinine levels were on average 1.9 mg/dL among COVID-19 patients who developed AKI, which is higher than the normal range values of 0.6–1.35 mg/dL (Table S2).

### 4.2. Predictors of AKI

**Figure 1:**
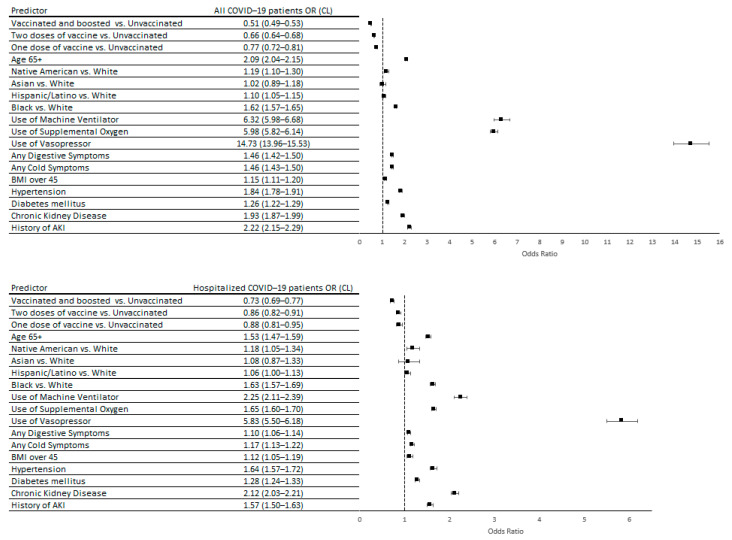
Predictors of developing AKI within 60 days after COVID-19 diagnosis among all patients and hospitalized patients at the US Department of Veterans Affairs between January 2020 and December 2022; N = 742,799. Hypertension, diabetes mellitus, chronic kidney disease, and history of AKI were defined as conditions recorded in the EHR in the 2 years prior to COVID-19 diagnosis.

The main predictors of developing AKI among COVID-19 patients (Figure 1) were use of vasopressors (OR = 14.73; 95% CL 13.96–15.53), history of AKI (OR = 2.22; CL 2.15–2.29), history of hypertension (OR = 1.84; CL 1.78–1.91), being male (OR = 1.90; CL 1.75–2.05), Black race (OR = 1.62; CL 1.57–1.65), or age over 65 years (OR = 1.57; CL 1.50–1.63). Receiving a COVID-19 vaccine before a COVID-19 diagnosis had a protective effect. Those who were vaccinated twice and boosted were least likely to develop AKI (OR = 0.51; CL 0.49–0.53) compared to unvaccinated COVID-19 patients. Similarly, double dose (OR = 0.77; CL = 0.72–0.81) and single dose (OR = 0.88; CL = 0.81–0.95) vaccinated patients were less likely to develop AKI compared to those who were not vaccinated. We found the same predictors of AKI among hospitalized COVID-19 patients (Figure 1).

### 4.3. Predictors of Mortality

**Figure 2:**
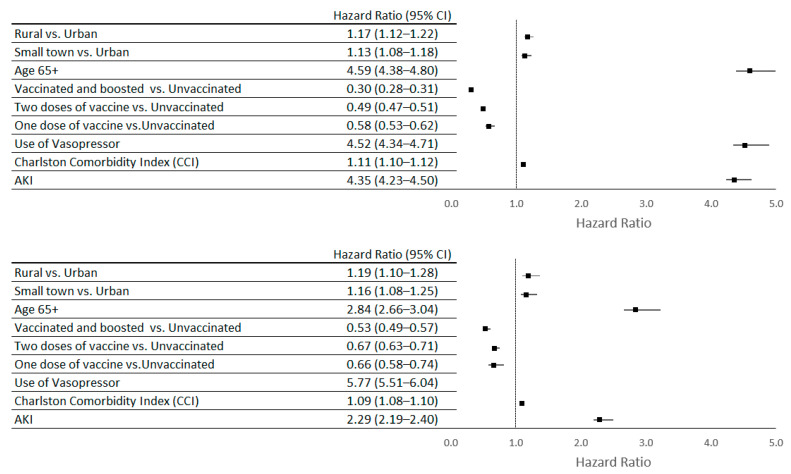
Predictors of 60-day mortality among all COVID-19 patients and hospitalized COVID-19 patients at the US Department of Veterans Affairs between January 2020 and December 2022; N = 742,799.

AKI was associated with a higher 60-day mortality risk for all COVID-19 patients (HR = 4.35; CL 4.23–4.50), as well as hospitalized patients (HR = 2.29; CL 2.19–2.40). Being vaccinated against COVID-19 showed a protective association, with the lowest mortality risk among the vaccinated and boosted patients (HR = 0.30; CL 0.28–0.31) (see Figure 2).

**Figure 3:**
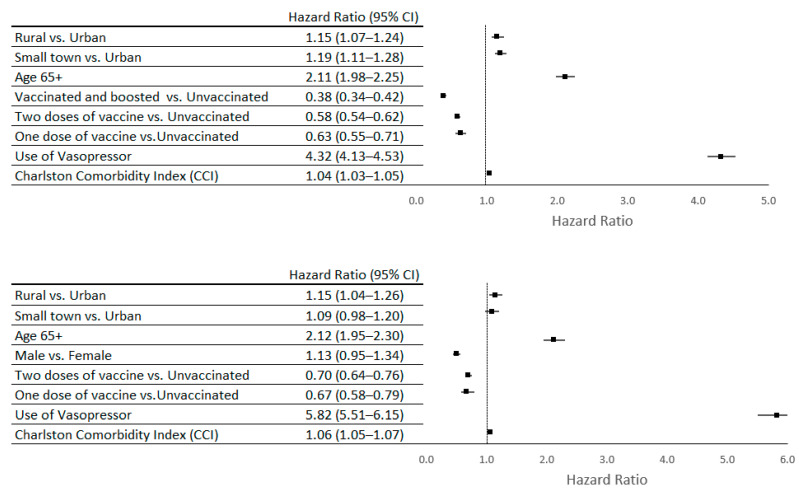
Predictors of 60-day mortality among COVID-19 AKI patients and hospitalized COVID-19 AKI patients at the US Department of Veterans Affairs between January 2020 and December 2022; N = 45,754.

The main predictors of 60-day mortality among AKI patients were the following: use of vasopressors (HR = 4.32; CL 4.13–4.53), age over 65 (HR =2.11; CL 1.98–2.25), and male gender (HR = 1.34; CL 1.17–1.53). AKI patients who were vaccinated (one or more doses of COVID-19 vaccine) had a lower 60-day mortality risk compared to unvaccinated AKI patients, where vaccinated and boosted patients had the lowest mortality risk (HR = 0.38; CL 0.34–0.42) (Figure 3). Similarly, we found the same predictors of mortality among AKI hospitalized patients (see Figure 3).

## 5. Discussion

Previous studies have illustrated that COVID-19 could contribute to AKI development, in most cases by indirect mechanisms such as extracellular volume depletion due to fever or GI symptoms (vomiting and diarrhea), and hypotension due to complications associated with sepsis or heart failure. Additionally, there was evidence that SARS-CoV-2 reduced kidney function by infecting kidney tissues and contributing to acute tubular injury and collapsing glomerulopathy [11,14,16,20,21,22,23,24,25].

Several studies with small sample sizes examined AKI among COVID-19 patients at the US Department of Veterans Affair (VA) during the early months of the pandemic [1,2,26]. Bowe et al. (2020) examined a cohort of VA patients who were hospitalized with COVID-19 by July 2020 and reported that developing AKI was associated with higher mechanical ventilator use, longer hospital stays, and increased mortality [2]. Additionally, Hung et al. (2022) examined a cohort of Black veterans hospitalized with COVID-19 between March 2020 and January 2021 and showed that having two copies of the APOL1 gene [26] variant was associated with higher odds of AKI, as well as increased severity and mortality.

To our knowledge, this is the only large study examining predictors of AKI using a nationwide cohort of US Veterans during the three years of the COVID-19 pandemic. Our study examined several groups of predictors of AKI development in COVID-19 patients. The first group of predictors included demographic characteristics such as older age, male gender, and belonging to a racial or ethnic minority group. The second group of predictors included comorbidities such as obesity, diabetes, hypertension, and history of AKI or chronic kidney disease (CKD). The last group of predictors included use of supplemental oxygen, mechanical ventilators, and vasopressors. Additionally, among hospitalized COVID-19 patients, lengths of hospital and ICU stays were examined. The study’s results, showing an association between these predictors with AKI development and mortality, were consistent with previous VA [1,2] and non-VA [4,5,9] studies.

Among COVID-19 patients, AKI was an independent predictor of mortality. The study results for predictors of mortality among COVID-19 patients with AKI were also consistent with previous research and showed that older age and use of vasopressors had the strongest association with higher mortality among this patient group [2,6]. In fact, use of vasopressors was the strongest predictor of both mortality and AKI development. A meta-analysis of 34 studies on the effect of vasopressors on mortality and AKI development among COVID-19 patients found the same association [27]. Since vasopressors are used to optimize mean arterial pressure (MAP), the association between use of vasopressors and mortality among COVID-19 patients could be due to excessive adrenergic stimulation, which might be a side effect of enhancement of the adrenergic pathway meant to improve hemodynamic support in patients with hypotension [27]. The association between use of vasopressors and AKI might be because these medications reduce kidney perfusion [28]. Additionally, use of vasopressors might be an indicator of acuity.

The main study finding was the lowered risk of developing AKI and mortality for patients who received the COVID-19 vaccination. More specifically, there was a decrease in mortality risk and AKI development for each additional vaccine dose; patients who received two doses of the COVID-19 vaccine followed by a booster had the lowest risk. To our knowledge, this is the only study that has shown the protective effect of COVID-19 vaccines against AKI development as well as mortality.

Although there were a considerable number of COVID-19 breakthrough infections among vaccinated individuals, mRNA vaccines were shown to be effective in preventing hospitalization and mortality among COVID-19 patients, especially among those who received a booster [29,30,31]. While it has previously been noted that older age, high BMI, and the presence of comorbidities can decrease the efficacy of COVID-19 vaccines [30], our study demonstrated that even patients with those characteristics were less likely to develop AKI or die if they were vaccinated. US military veterans in general, including the majority of our study cohort, are on average older and sicker than the US general population, and therefore they were most likely to experience the lower efficiency of the vaccines compared to the general patient populations. Nevertheless, we observed a strong inverse association between the number of vaccines and risk of death or AKI development, which might suggest that this effect could be even stronger among younger and healthier individuals.

Other studies have shown that negative attitudes about seasonal flu vaccines, concerns about vaccine safety, distrust in authorities, conspiracy beliefs, and previous COVID-19 infections were some of the barriers to vaccination in the United States [32,33]. While there were no previous studies examining vaccine hesitancy among AKI patients at the VA, a study conducted among VA users in 2021 found that White race, female gender, living in urban areas, having more comorbidities, and a history of receiving seasonal flu vaccine were associated with receiving at least one dose of COVID-19 vaccine [34]. At the same time, another VA study on home-based primary care VA users reported that lack of transportation, difficulty scheduling an appointment for vaccination, and lack of educational resources among healthcare providers were perceived as barriers to receiving COVID-19 vaccines [35]. Moreover, when it came to making decisions about getting vaccinated, US veterans showed that unique factors such as adherence to military culture as well as trusting veteran peers and individual healthcare providers as opposed to trusting the entire healthcare system [36,37] were influential.

Our study has several limitations. As noted, US veterans tend to differ from the general US population [38,39] (older, sicker, lower socio-economic status, mostly men) and therefore the results might not be generalizable to the general population. Additionally, we did not have data to capture AKI cases that developed 60 days after COVID-19 diagnosis. However, it is important to note that beyond 60 days from the initial infection, it is less likely for the development of AKI to be associated with the COVID-19 diagnosis. Additionally, we did not examine the different types of COVID-19 vaccines, since the focus of the study was on examining the number of COVID-19 vaccinations that patients had received before COVID-19 diagnosis. Future studies should examine the effect of different COVID-19 vaccines (e.g., mRNA vaccines vs. other types of vaccines) on developing AKI or risk of death among COVID-19 patients.

The strengths of our study include using a large nationwide cohort of US veterans during a three-year study period, which allowed us to examine a large number of COVID-19-positive patients who developed AKI. Also, we used electronic heath records from an integrated healthcare system, which allowed us to ascertain comorbidities, baseline lab results, and vaccination status.

## 6. Conclusions

Our study not only examines predictors of AKI among COVID-19 patients, but also indicates the significant protective role of COVID-19 vaccinations in decreasing the risk of death and developing AKI. This study contributes to the growing knowledge that older age, history of CKD or AKI, male gender, use of vasopressors, mechanical ventilators, and supplemental oxygen are associated with both the development of AKI and a higher risk of mortality among COVID-19 AKI patients. Additionally, our study underscores the importance of COVID-19 vaccination as an important factor in reducing the overall burden of the disease on healthcare systems as vaccination contributed to reductions in both COVID-19 complications and mortality. Accordingly, this study lends additional support to the importance of developing organizational and government policies that effectively promote COVID-19 vaccination, including boosters, among at-risk population groups. Our finding that each vaccine dose was increasingly protective is particularly important given the relatively low rates of booster vaccine uptake reported by the Centers for Disease Control and Prevention. Moreover, it adds to the growing literature establishing the important role of vaccines, including boosters, in preventing the potential complications, including death, of COVID-19 among veterans and other populations.

## Figures and Tables

**Table 1:** Characteristics of COVID-19 patients at the US Department of Veterans Affairs between January 2020 and December 2022 by AKI and hospitalization status; N = 742,799.

Characteristics	AKI No. (%)	Non-AKI No. (%)	*p*-Value	AKI No. (%)	Non-AKI No. (%)	*p*-Value
All patients				Hospitalized patients		
Number of patients	45,754	697,045		28,573	67,000	
All deaths	15,747 (34)	39,712 (6)	<0.0001	10,536 (37)	11,188 (17)	<0.0001
Comorbidities in the past 2 years prior COVID-19 diagnosis						
History of AKI	11,042 (24)	33,033 (5)	<0.0001	7536 (26)	9646 (14)	<0.0001
History of dialysis	466 (1)	1769 (0.3)	<0.0001	325 (1)	403 (0.6)	<0.0001
Chronic kidney disease	16,468 (36)	62,469 (9)	<0.0001	10,579 (37)	11,426 (17)	<0.0001
Diabetes mellitus	25,518 (54)	184,021 (26)	<0.0001	15,399 (54)	26,465 (40)	<0.0001
Any cardiovascular disease (any CVD)	26,124 (57)	187,552 (27)	<0.0001	17,138 (60)	32,974 (49)	<0.0001
Heart failure	11,516 (25)	46,114 (7)	<0.0001	7881 (28)	11,539 (17)	<0.0001
Pulmonary heart disease	2514 (5)	9891 (2)	<0.0001	1794 (5)	2791 (4)	<0.0001
Chronic lung disease	18,744 (41)	174,276 (25)	<0.0001	12,232 (43)	26,658 (40)	<0.0001
Acute respiratory failure	5934 (5)	25,665 (1)	<0.0001	3993 (13)	7239 (11)	<0.0001
Chronic obstructive pulmonary disease (COPD)	12,624 (28)	86,523 (12)	<0.0001	8325 (29)	17,513 (26)	<0.0001
Hypertension	38,096 (83)	353,983 (51)	<0.0001	24,121 (84)	47,534 (71)	<0.0001
Never smoker	14,782 (35)	241,802 (42)	<0.0001	8973 (34)	21,326 (35)	<0.0001
Former smoker	20,773 (49)	240,948 (42)	<0.0001	12,761 (49)	27,603 (45)	<0.0001
Current smoker	6703 (15.8)	92,002 (16.0)	<0.0001	4355 (17)	12,090 (20)	<0.0001
BMI over 45	3693 (8)	48,637 (7)	<0.0001	2094 (8)	4502 (7)	0.001
Charlson comorbidity index (CCI) ≥ 5	13,385 (29)	49,999 (7)	<0.0001	9411 (33)	13,346 (20)	<0.0001
Symptoms on a day or up to 30 days before COVID-19 diagnosis						
Dyspnea	16,861 (36)	130,505 (18)	<0.0001	13,033 (46)	26,969 (40)	<0.0001
Any cold symptoms	27,074 (59)	291,294 (42)	<0.0001	19,695 (69)	42,584 (64)	<0.0001
Any digestive symptoms	12,286 (27)	107,423 (15)	<0.0001	9145 (32)	19,097 (29)	<0.0001
Fever	15,081(33)	141,499 (20)	<0.0001	11,289 (40)	24,264 (36)	<0.0001
Nausea	6732 (15)	59,327 (9)	<0.0001	5029 (18)	11,007 (16)	<0.0001
Diarrhea	7601 (17)	66,333 (10)	<0.0001	5632 (20)	10,571 (17)	<0.0001
COVID-19 complications within 60 days of COVID-19 diagnosis						
Emergency department visits within 60 days of COVID-19 diagnosis	32,896 (72)	241,577 (35)	<0.0001	26,046 (91)	57,578 (86)	<0.0001
Hospital readmission within 30 days from discharge	5076 (11)	6401 (1)	<0.0001	5076 (18)	6401 (10)	<0.0001
Use of vasopressor	5078 (11)	3375 (0.5)	<0.0001	4975 (17)	2267 (3)	<0.0001
ICU admission/transfer	12,531 (28)	15,053 (2)	<0.0001	12,531 (44)	15,053 (23)	<0.0001
Use of supplemental oxygen	20,248 (45)	36,471 (5)	<0.0001	20,207 (72)	36,087 (55)	<0.0001
Use of machine ventilator	5900 (13)	5063 (1)	<0.0001	5273 (19)	3420 (5)	<0.0001
Any acute respiratory failure	21,412 (47)	38,682 (6)	<0.0001	15,258 (54)	24,089 (36)	<0.0001
Dialysis within 60 days of COVID-19 diagnosis	1998(5)	0		1669 (6)	0	
Chronic kidney failure (CKF) 60 days	1394 (3)	96 (0.1)	<0.0001	1107 (4)	64 (0.1)	<0.0001
Demographic characteristics						
White	26,980 (62)	394,455 (66)	<0.0001	16,253 (60)	42,506 (67)	<0.0001
Black	12,253 (28)	127,608 (21)	<0.0001	8269 (30)	14,282 (22)	<0.0001
Hispanic/Latino	3360 (8)	61,559 (10)	<0.0001	2133 (8)	5563 (9)	<0.0001
Asian	274 (0.6)	7997 (1)	<0.0001	168 (0.6)	465 (0.7)	<0.0001
Native American	715 (1.6)	9929 (1.5)	<0.0001	421 (1.5)	958 (1.5)	<0.0001
Males	43,841 (96)	562,173 (81)	<0.0001	27,458 (96)	61,992 (93)	<0.0001
Married	22,169 (49)	335,804 (53)	<0.0001	13,221 (47)	30,227 (45)	0.5
Age 65+	33,392 (73)	270,649 (39)	<0.0001	21,510 (75)	41,055 (61)	<0.0001
Year 2020	12,725 (28)	154,376 (22)	<0.0001	8431 (30)	17,204 (27)	<0.0001
Year 2021	16,165 (35)	233,454 (33)	<0.0001	9728 (34)	22,711 (34)	<0.0001
Year 2022	16,864 (37)	309,215 (44)	<0.0001	10,414 (36)	27,085 (40)	<0.0001
Vaccination status						
Unvaccinated before COVID-19 diagnosis	28,130 (61)	420,794 (60)	<0.0001	17,713 (62)	39,831 (59)	<0.0001
One COVID-19 vaccine before COVID-19 diagnosis	1921 (4.2)	30,170 (4.3)	0.0003	1164 (4)	3039 (5)	<0.0001
Two doses of COVID-19 vaccine before COVID-19 diagnosis	8388 (18)	131,568 (19)	<0.0001	5136 (18)	12,426 (19)	0.001
Vaccinated and boosted before COVID-19 diagnosis	7315 (15.9)	114,513 (16.5)	0.03	4508 (16)	11,704 (1)	<0.0001

**Table 2:** Indicators of hospitalization among COVID-19 patients at the US Department of Veterans Affairs between January 2020 and December 2022 by AKI status; N = 95,573.

Hospitalized COVID-19 pt.	AKI Patients N = 28,573	Mean/Median (SD)	Non-AKI Patients N = 67,000	Mean/Median (SD)
Age at the time of COVID-19 diagnosis	28,571	71/72 (12.3)	66,967	66/69 (15.1)
CCI in the past 2 years	28,573	3.6/3 (2.8)	67,000	2.6/2 (2.6)
BMI	28,508	29/28 (7.3)	66,811	29/28 (7.0)
Hospital length of stay (LOS) in days	26,791	11.9/8 (13.2)	58,984	7.5/4 (11.0)
ICU length of stay (ICULOS) in days	13,784	9.1/6 (10.3)	19,082	5.6/4 (6.9)
Acute care length of stay (ACLOS) in days	22,337	8.7/5 (11.0)	51,451	6.5/4 (10.5)
Number of days between COVID-19 diagnosis and hospital admission	27,561	3.9/0 (10.2)	60,917	6/0 (12.9)
Number of days between COVID-19 diagnosis and ICU admission	12,349	7.1/1 (11.9)	14,644	7.7/1 (13.2)
Number of days on a ventilator	5240	10.8/6 (27.6)	3406	8.1/1 (33.9)

## Data Availability

The study data belongs to the US Department of Veterans Affairs. Deidentified table counts can be provided upon request.

## Supplementary Materials

The following are available online at https://www.mdpi.com/article/10.3390/vaccines12020146/s1, Figure S1: Monthly numbers of new COVID-19 cases that developed AKI within 60 days of COVID-19 diagnosis among VA patients between January 2020 and December 2022; Table S1: Additional Clinical and demographic characteristics of COVID-19 patients at the US Department of Veterans Affairs between January 2020 and December 2022 by AKI and hospitalization status; Table S2: Baseline blood level values for laboratory parameters associated with kidney disease and inflammation among hospitalized COVID-19 patients at US Department of Veterans Affairs between January 2020 and December 2022 by AKI status; N = 95,573.
