# Supplementary material for: Predictors of Acute Kidney Injury (AKI) among COVID-19 Patients at the US Department of Veterans Affairs: The Important Role of COVID-19 Vaccinations
Source: Vaccines (Basel). 2024 Jan 30;12(2):146. doi: 10.3390/vaccines12020146 (PMC10892207; doi:10.3390/vaccines12020146)
Supplement: Supplementary file 1 [file vaccines-12-00146-s001.zip › vaccines-2790291-supplementary.pdf]

Supplement

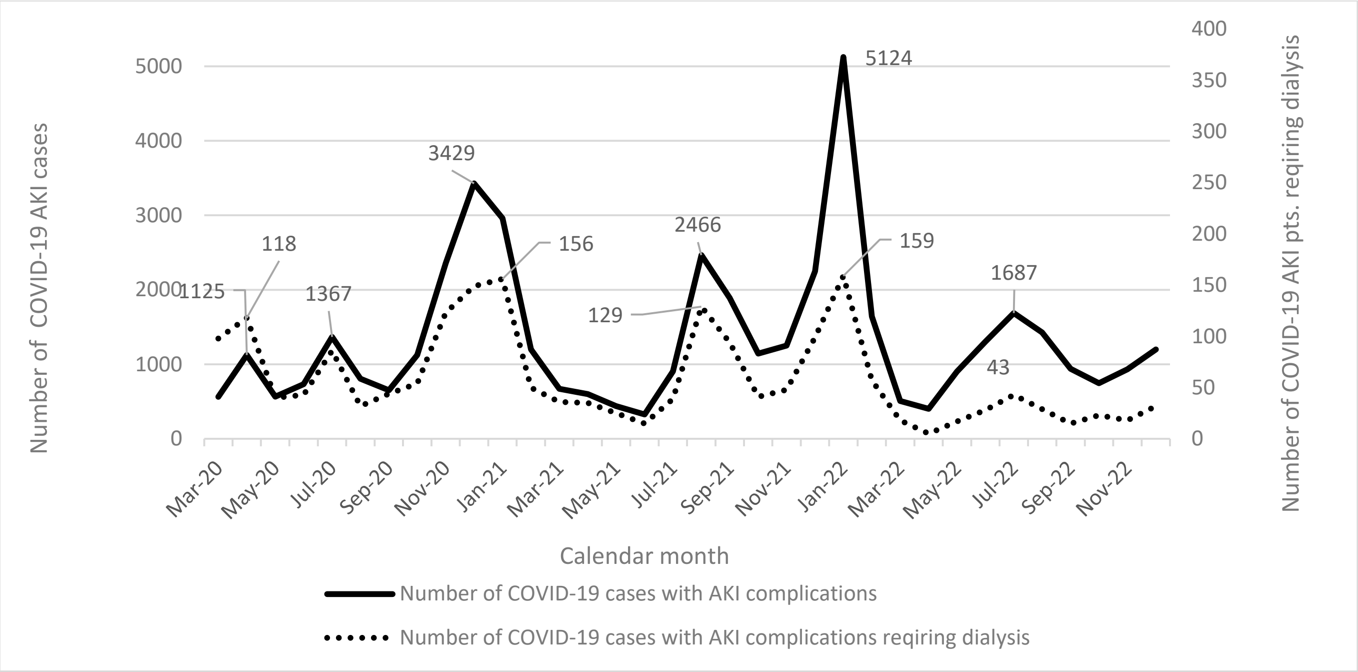

Figure S1. Monthly numbers of new COVID-19 cases that developed AKI within 60 days of COVID-19 diagnosis among VA patients between January 2020 and December 2022

Table S1. Additional Clinical and demographic characteristics of COVID-19 patients at the US Department of Veterans Affairs between January 2020 and December 2022 by AKI and hospitalization status.

Characteristics	AKI No. (%)	no AKI No. (%)	p-value	AKI No. (%)	no AKI No. (%)	p-value
All patients				***Hospitalized patients***		
Number of patients	45,754	697,045		28,573	67,000	
30-day Mortality	8,234 (18)	11,760 (2%)	<.0001	5,160 (18)	3,080 (5)	<.0001
60-day mortality	10,338(23)	15,330 (2)	<.0001	6,686 (23)	4,390 (7)	<.0001
Comorbidities in the past 2 years period COVD1-19 diagnosis						
Nephrosis	3,342 (7)	11,003 (1)	<.0001	2185 (8)	2,055 (3)	<.0001
Any kidney disease	22,160 (48)	113,011 (16)	<.0001	14,371 (50)	20,595 (31)	<.0001
Urinary Stones	7,692 (17)	51,790 (7)	<.0001	5,039 (18)	8,569 (13)	<.0001
Cardiomyopathy	3,841 (8)	16,594 (2)	<.0001	2,661 (9)	3,880 (6)	<.0001
Arrhythmia	2,732 (6)	19,831 (3)	<.0001	1,972 (7)	3,957 (6)	<.0001
Acute Cardiac Injury (ACI)/Acute MI	2,892 (6)	12,031 (2)	<.0001	1,997 (7)	3,108 (5)	<.0001
Coronary Atherosclerotic Disease (CAHD)	16,028 (35)	108,370 (16)	<.0001	10,397 (36)	19,215 (29)	<.0001
Peripheral Arterial Disease (PAD)	10,134 (22)	52,933 (8)	<.0001	6,856 (24)	11,565 (17)	<.0001
Venous Thromboembolism (VTE)	2,347 (5)	14,102 (2)	<.0001	1,633 (6)	3,200 (5)	<.0001
Cerebrovascular Disease	19,23 (4)	10,897 (2)	<.0001	1,377 (5)	2,731 (4)	<.0001
Pneumonia	5,834(13)	29,580 (4)	<.0001	3,961 (14)	7,691 (11)	<.0001
Bronchitis	3,236 (7)	32,515 (5)	<.0001	2,179 (8)	5,112 (8)	0.3
Lower Respiratory Tract Infection	3,620 (8)	45,048 (7)	<.0001	2,378 (8)	5,553 (8)	0.9
Any Respiratory Disease	23,108 (51)	226,531 (33)	<.0001	14,915 (52)	32,947 (49)	<.0001
Obstructive Sleep Apnea (OSA)	14,954 (33)	200,262 (29)	<.0001	9,148 (32)	21174 (32)	0.2
Hyperlipidemia	32,247 (70)	355,017 (51)	<.0001	20,022 (70)	42,670 (64)	<.0001
Liver Disease	4,481 (10)	41,736 (6)	<.0001	3,078 (11)	6,757 (10)	0.001
Septic Shock	34,92 (8)	12,909 (2)	<.0001	2,422 (8)	4,100 (6)	<.0001
Cancer	10,842 (24)	79,332 (11)	<.0001	7,218 (25)	14,287 (21)	<.0001
PTSD	9,468 (21)	163,318 (23)	<.0001	5,727 (20)	17,215 (26)	<.0001
Major Depressive Disorder (MDD)	14,435 (32)	216,262 (31)	0.1	9,060 (32)	25,576 (38)	<.0001
Anxiety disorder	8,349 (18)	148,388 (21)	<.0001	5,144 (18)	16,403 (24)	<.0001
Alcohol dependency	8,133 (18)	130,603 (19)	<.0001	5,286 (19)	15,822 (24)	<.0001

Characteristics	AKI No. (%)	no AKI No. (%)	p-value	AKI No. (%)	no AKI No. (%)	p-value
Drug dependency	2,751 (6)	29,210 (4)	<.0001	1,914 (7)	6,786 (10)	<.0001
Any Mental Health Encounters	23,211 (51)	354,843 (51)	0.5	14,450 (51)	38,846 (58)	<.0001
Primary care visit(s) during 18 months before COVID-19 diagnosis	43,548 (95)	595,482 (85)	<.0001	26,961 (94)	62,672 (93)	<.0001
Symptoms on a day or up to 30 days before COVID-19 diagnosis						
Cough	16,525 (36)	194,503 (28)	<.0001	11,779 (41)	26,315 (39)	<.0001
Fatigue	11,077 (24)	88,660 (13)	<.0001	8,439 (30)	16,582 (26)	<.0001
Chills	3,880 (8)	57,273 (8)	0.05	2,509 (9)	6,082 (9)	0.1
Myalgia	3,183 (7)	58,986 (9)	<.0001	1,918 (7)	5,066 (8)	<.0001
Headache	5,275 (12)	102,183 (15)	<.0001	3,287 (12)	9,524 (10)	<.0001
Loss of Smell or Taste	2,544 (6)	40,423 (6)	0.03	1,718 (6)	4,120 (6)	0.4
Sore Throat	3,101 (7)	72,115 (10)	<.0001	1,792 (6)	4,814 (7)	<.0001
Abdominal pain	3,007 (7)	22,862 (3)	<.0001	2,357 (8)	5,658 (7)	0.3
COVID-19 complications within 60 days of COVID-19 diagnosis						
Hospital Acquired VTE	408 (1)	296 (0.4)	<.0001	408 (1.5)	296(0.5)	<.0001
Acute Respiratory Failure	20,375 (45)	37,748 (5)	<.0001	15,084 (53)	23,836 (36)	<.0001
Respiratory Distress Syndrome (ARDS)	3,649 (8)	2,425 (0.4)	<.0001	2,485 (9)	1,461 (2)	<.0001
Acute Myocardial Infarction (MI)	4,287 (10)	4,901 (1)	<.0001	3,214 (11)	3,081 (5)	<.0001
Arrhythmia	1,830 (4)	5,469 (1)	<.0001	1,647 (6)	2,747 (4)	<.0001
Peripheral Arterial Disease (PAD)	5,829/ (13)	16,340 (2)	<.0001	4,680 (17)	7,187 (11)	<.0001
VTE	3,894/ (9)	9,605 (1)	<.0001	2,957 (11)	4,626 (7)	<.0001
Blood Transfusion	4,174 (9)	3,485 (0.5)	<.0001	4,104/ (15)	3,316 (5)	<.0001
Demographic characteristics						
Urban	31,972 (83)	498,269 (84)	<.0001	20,094 (85)	44,130 (85)	0.03
Small Town	3,452 (9)	52,600 (9)	<.0001	1,846 (8)	4,171 (8)	0.03
Rural	3,045 (8)	40,124 (7)	<.0001	1,667 (7)	3,627 (7)	0.03
North-Eastern Region	10,006 (22)	156,605 (23)	<.0001	6,060 (21)	13,989 (21)	0.03
Mid-Western Region	10,268 (23)	159,601 (23)	<.0001	6,537 (23)	15,942 (24)	0.03
Southern Region	12,850 (28)	185,671 (28)	<.0001	8,076 (28)	18,313 (28)	0.03
South-Central Region	4,955 (10)	72,407 (11)	<.0001	3,187 (11)	7,311 (11)	0.03

Characteristics	AKI No. (%)	no AKI No. (%)	p-value	AKI No. (%)	no AKI No. (%)	p-value
Western Region	7,364 (16)	116,786 (16)	<.0001	4,549 (16)	10,867 (16)	0.03

Table S2. Baseline blood level values for laboratory parameters associated with kidney disease and inflammation among hospitalized COVID-19 patients at US Department of Veterans Affairs between January 2020 and December 2022 by AKI status; N=95,573

**Blood level**	**AKI patients N=28,573**	**Mean/Median (SD)**	**non-AKI patients N=67,000**	**Mean/Median (SD)**	**Normal ranges**
Albumin (g/dL)	24,876	3.5 /3.5 (0.7)	59,197	3.6/3.7 (0.6)	3.4-5.4
BUN (mg/dL)	25,273	5.1/3.1 (7.8)	54,250	2.9/1.7 (4.7)	6.0-24
Creatinine (mg/dL)	26,698	1.9/1.6 (1.8)	58,550	1.1/1.0 (0.3)	0.6-1.35
C-reactive protein (mg/L)	15,128	91/68 (87)	29,231	63/41 (62)	under 10
Ferritin (ng/mL)	16,141	221/117 (246)	29,822	197/94 (231)	24-336
eGFR (ml/min/1.73m^2)	21,787	32/24 (32)	41,532	50/56 (41)	over 60
Hemoglobin (g/dL)	25,982	12.3 /12.5 (2.5)	57,189	13.2 /13.4 (2.2)	13.8-17.2
LDH (IU/L)	12,918	217/158 (305)	23,820	160/111 (180)	105-333
Lymphocytes (%)	18,940	15/13 (11)	39,859	19/17 (12)	20-40
Neutrophils (%)	17,700	73 /75 (12)	37,680	67/70 (17)	40-60
Platelets (X10^9/L)	26,078	143/135 (128)	57,681	143 /145 (128)	150-400
WBC (X10^9/L)	26,557	8.9/7.4 (9)	58,354	7.7/6.8 (6.3)	4.5-11
